# Supplementary material for: Risk Factors for Failure of Direct Oral Feeding Following a Totally Minimally Invasive Esophagectomy
Source: Nutrients. 2021 Oct 15;13(10):3616. doi: 10.3390/nu13103616 (PMC8539606; doi:10.3390/nu13103616)
Supplement: Supplementary file 1 [file nutrients-13-03616-s001.zip › nutrients-1310641-supplementary.pdf]

Supplementary File I

Supplementary Table S1. Patient characteristics by adherence to feeding regimen: Subgroup analysis for any complication.

	**No Deviation**		**Deviation Due to Complications**		
	n = 116		n = 31		*p*-value
**Age**, years	65	[9]	64	[7]	0.769
**BMI**, kg/m2	26.1	[23.4-29.3]	25.6	[23.9-29.7]	0.987
**Weight loss**, kg	0	[0-5]	2	[0-6]	0.744
**Sex**, male	99	(85.3)	23	(74.2)	0.142
**ASA**					**0.043**
II	86	(74.1)	17	(54.8)	
III	27	(23.3)	14	(45.2)	
IV	3	(2.6)	0		
**Smoking history**	92	(79.3)	27	(87.1)	0.327
Active smoker (or quit <1 year)	33	(28.7)	12	(38.7)	0.284
**Alcohol consumption**					0.920
Daily	38	(33.0)	11	(35.5)	
Weekly	18	(15.7)	4	(12.9)	
**Preoperative Tube feeding**	25	(21.6)	10	(32.3)	0.214
**Comorbidity**	74	(63.8)	21	(67.7)	0.683
Cardiac	17	(14.7)	5	(16.1)	0.838
Pulmonary	23	(19.8)	7	(22.6)	0.735
COPD	13	(11.2)	5	(16.1)	0.458
Vascular	40	(34.5)	13	(41.9)	0.443
Hypertension	36	(31.0)	12	(38.7)	0.418
Diabetes	15	(12.9)	4	(12.9)	0.997
Obesity	25	(21.6)	7	(22.6)	0.902
**(c)TNM stage**					**0.017**
Stage 0	1	(0.9)	0		
Stage I	4	(3.4)	6	(19.4)	
Stage II	22	(19.0)	7	(22.6)	
Stage III	61	(52.6)	15	(48.4)	
Stage IV	28	(24.1)	3	(9.7)	

Legend: values are absolute numbers (percentage) or medians [lower quartile – upper quartile] means [standard deviation]. BMI body mass index; ASA American Society of Anesthesiologists; (c)TNM clinical TNM staging.

Supplementary Table S2. Patient characteristics by adherence to feeding regimen: subgroup analysis for insufficient intake.

	**No Deviation** n = 116		**Deviation Due to Insufficient Intake** n = 18		*p*-value
**Age**, years	65	[9]	64	[9]	0.595
**BMI**, kg/m2	26.1	[23.4-29.3]	24.8	[23.2-29.1]	0.395
**Weight loss**, kg	0	[0-5]	4	[0-8]	0.275
**Sex**, male	99	(85.3)	9	(50.0)	**<0.001**
**ASA**					
II	86	(74.1)	15	(83.3)	0.624
III	27	(23.3)	3	(16.7)	
IV	3	(2.6)	0		
**Smoking history**	92	(79.3)	13	(72.2)	0.497
Active smoker (or quit <1 year)	33	(28.7)	5	(27.8)	0.936
**Alcohol consumption**					0.229
Daily	38	(33.0)	4	(22.2)	
Weekly	18	(15.7)	1	(5.6)	
**Preoperative Tube feeding**	25	(21.6)	6	(33.3)	0.270
**Comorbidity**	74	(63.8)	11	(61.1)	0.826
Cardiac	17	(14.7)	3	(16.7)	0.824
Pulmonary	23	(19.8)	4	(22.2)	0.814
COPD	13	(11.2)	2	(11.1)	0.990
Vascular	40	(34.5)	4	(22.2)	0.303
Hypertension	36	(31.0)	4	(22.2)	0.447
Diabetes	15	(12.9)	0		0.105
Obesity	25	(21.6)	3	(16.7)	0.635
**(c)TNM stage**					0.082
Stage 0	1	(0.9)	1	(5.6)	
Stage I	4	(3.4)	3	(16.7)	
Stage II	22	(19.0)	2	(11.1)	
Stage III	61	(52.6)	8	(44.4)	
Stage IV	28	(24.1)	4	(22.2)	

Legend: values are absolute numbers (percentage) or medians [lower quartile – upper quartile] means [standard deviation]. BMI body mass index; ASA American Society of Anesthesiologists; (c)TNM clinical TNM staging.

Supplementary Table S3. Patient characteristics by anastomotic leakage.

	Uncomplicated		Anastomotic Leakage		
	n = 77		n = 15		*p*-value
**Age**, years	65	[56-71]	60	[56-66]	0.186
**BMI**, kg/m2	26.0	[23.7-29.9]	25.7	[23.9-33.0]	0.845
**Weight loss**, kg	0	[0-5]	3	[2-6]	0.158
**Sex**, male	64	(83.1)	13	(86.7)	0.733
**ASA**					**0.001**
II	59	(76.6)	5	(33.3)	
III	18	(23.4)	10	(66.7)	
**Smoking history**	58	(75.3)	15	(100)	**0.031**
Active smoker (or quit <1 year)	14	(18.4)	6	(40.0)	0.065
**Alcohol consumption**					0.611
Daily	21	(27.3)	6	(40.0)	
Weekly	13	(16.9)	2	(13.3)	
**Preoperative Tube feeding**	15	(19.5)	5	(33.3)	0.234
**Comorbidity**	49	(63.6)	12	(80.0)	0.220
Cardiac	9	(11.7)	3	(20.0)	0.382
Pulmonary	17	(22.1)	4	(26.7)	0.698
COPD	8	(10.4)	3	(20.0)	0.294
Vascular	24	(31.2)	7	(46.7)	0.245
Hypertension	23	(29.9)	6	(40.0)	0.440
Diabetes	11	(14.3)	2	(13.3)	0.923
Obesity	17	(22.1)	4	(26.7)	0.698
**(c)TNM stage**					0.444
Stage 0	1	(1.3)	0		
Stage I	5	(6.5)	2	(13.3)	
Stage II	12	(15.6)	4	(26.7)	
Stage III	40	(51.9)	8	(53.3)	
Stage IV	19	(24.7)	1	(6.7)	

Legend: values are absolute numbers (percentage) or medians [lower quartile – upper quartile] means [standard deviation]. BMI body mass index; ASA American Society of Anesthesiologists; (c)TNM clinical TNM staging.

Supplementary Table S4. Patient characteristics by pneumonia (UPS).

	**Uncomplicated** n = 77	**Pneumonia** n = 36	*p*-value
**Age**, years	63 [10]	65 [7]	0.190
**BMI**, kg/m2	26.8 [4.4]	26.4 [4.7]	0.664
**Weight loss**, kg	0 [0-5]	2 [0-6]	0.498
**Sex**, male	64 (83.1)	9 (25.0)	0.310
**ASA**			**0.023**
II	59 (76.6)	20 (55.6)	
III	18 (23.4)	16 (44.4)	
**Smoking history**	58 (75.3)	34 (94.4)	**0.015**
Active smoker (or quit <1 year)	14 (18.4)	19 (52.8)	**<0.001**
**Alcohol consumption**			**0.030**
Daily	21 (27.3)	17 (47.2)	
Weekly	13 (16.9)	1 (2.8)	
**Preoperative Tube feeding**	15 (19.5)	12 (33.3)	0.108
**Comorbidity**	49 (63.6)	26 (72.2)	0.368
Cardiac	9 (11.7)	5 (13.9)	0.741
Pulmonary	17 (22.1)	8 (22.2)	0.986
COPD	8 (10.4)	8 (22.2)	0.093
Vascular	24 (31.2)	15 (41.7)	0.274
Hypertension	23 (29.9)	11 (30.6)	0.941
Diabetes	11 (14.3)	3 (8.3)	0.371
Obesity	17 (22.1)	8 (22.2)	0.986
**(c)TNM stage**			0.549
Stage 0	1 (1.3)	0	
Stage I	5 (6.5)	3 (8.3)	
Stage II	12 (15.6)	10 (27.8)	
Stage III	40 (51.9)	15 (41.7)	
Stage IV	19 (24.7)	8 (22.2)	

Legend: values are absolute numbers (percentage) or medians [lower quartile – upper quartile] means [standard deviation]. BMI body mass index; ASA American Society of Anesthesiologists; (c)TNM clinical TNM staging.

Supplementary Table S5. Patient characteristics by histological subtype.

	**AC and Other** n = 145	**SCC** n = 20	*p*-value
**Age**, years	64 [9]	66 [7]	0.304
**BMI**, kg/m2	26.0 [23.5-29.8]	24.6 [22.8-27.6]	0.226
**Weight loss**, kg	1.0 [0-6.0]	1.5 [0-5.8]	0.934
**Sex**, male	119 (82.1)	12 (60.0)	**0.022**
**ASA**			0.265
II	102 (70.3)	16 (80.0)	
III	41 (28.3)	3 (15.0)	
IV	2 (1.4)	1 (5.0)	
**Smoking history**	115 (79.3)	17 (85.0)	0.551
Active smoker (or quit <1 year)	44 (30.6)	6 (30.0)	0.960
**Alcohol consumption**			0.093
Daily	43 (29.7)	10 (52.6)	
Weekly	20 (13.8)	3 (15.8)	
**Preoperative tube feeding**	35 (24.1)	6 (30.0)	0.570
**Comorbidity**	96 (66.2)	10 (50.0)	0.156
Cardiac	21 (14.5)	4 (20.0)	0.519
Pulmonary	31 (21.4)	3 (15.0)	0.508
COPD	17 (11.9)	3 (15.0)	0.803
Vascular	53 (36.6)	3 (15.0)	0.056
Hypertension	49 (34.3)	3 (15.0)	0.139
Diabetes	19 (13.1)	0	0.085
Obesity	33 (22.8)	2 (10.0)	0.191
**(c)TNM stage**			**0.026**
0	2 (1.4)	0 -	
I	9 (6.2)	4 (20.0)	
II	24 (16.6)	7 (35.0)	
III	76 (52.4)	8 (40.0)	
IV	32 (23.4)	1 (5.0)	
**Surgical lymph node yield**	27 [21-32]	29 [21-33]	0.740

Legend: values are absolute numbers (percentage) or medians [lower quartile – upper quartile] means [standard deviation]. BMI body mass index; ASA American Society of Anesthesiologists; (c)TNM clinical TNM staging.
